# A day-to-day management model improves patient compliance to treatment for *Helicobacter pylori* infection: a prospective, randomized controlled study

**DOI:** 10.1186/s13099-023-00556-x

**Published:** 2023-07-31

**Authors:** Zhen Yang, Wenjie Xiong, Ruoyun Yang, Haisheng Qian, Zhi He, Meihong Chen, Jiajia Yang, Huaiming Sang, Jin Yan, Xiaobing Xu, Yun Wang, Guoxin Zhang, Feng Ye

**Affiliations:** 1 Department of Gastroenterology, First Affiliated Hospital of Nanjing Medical University, No.300 Guangzhou Road, Nanjing, 210029 Jiangsu Province China; 2 First Clinical Medical College of Nanjing Medical University, Nanjing, 210029 Jiangsu Province China

**Keywords:** *Helicobacter pylori* infection, Day-to-day, Conventional outpatient education, Habit formation, Compliance

## Abstract

**Background:**

The day-to-day (DTD) management model encourages patients to actively participate in their healthcare by setting goals. We determined the effectiveness of the DTD model in the treatment of *Helicobacter pylori* (*H. pylori*) infection, as compared with conventional outpatient education (OE).

**Methods:**

We randomized 254 *H. pylori*-positive patients into a DTD group (127 patients) and an OE group (127 patients) prior to primary treatment with 14-day bismuth-containing quadruple therapy, including esomeprazole, amoxicillin, and clarithromycin. Both groups received consistent medication instructions. Patients in the DTD group recorded daily attendance after completing their daily medication plan from day 1 to day 14. The medication compliance, follow-up compliance, *H. pylori* eradication rates, and adverse events (AEs) were evaluated.

**Results:**

In the modified intention-to-treat (MITT) and per-protocol (PP) analyses, the DTD group showed significantly higher medication compliance than the OE group (*P* = 0.001 and *P* = 0.031, respectively). Both the MITT and PP analyses showed significant differences in follow-up compliance (*P* < 0.001 and *P* = 0.003, respectively) and timing of the review urea breath test (*P* < 0.001 and *P* = 0.001, respectively) between the two groups. However, no significant differences were observed in the *H. pylori* eradication rates (95.8% vs. 93.8%, P = 0.529) in the PP analysis, or AEs incidence (25.4% vs. 28.3%, P = 0.603) between the two groups.

**Conclusion:**

This study demonstrated the novel application of the DTD model in the treatment of *H. pylori* infection, which enabled patients to develop habitual medication-taking behaviors without physician intervention.

**Supplementary Information:**

The online version contains supplementary material available at 10.1186/s13099-023-00556-x.

## Background

*Helicobacter pylori* infection increases the risk of peptic ulcer disease and gastric cancer [1]. *H. pylori* infection affects approximately 50% of the world’s population [2], and the average family-based infection rate in China is 71.2% [3]. *H. pylori* eradication can reduce the incidence and mortality of gastric cancer, and might confer long-term protection against gastric cancer in high-risk populations [4]. Hence, effective management resulting in *H. pylori* eradication is essential to prevent harmful outcomes.

Poor compliance is a major predictor of *H. pylori* treatment failure, and can contribute to antibiotic resistance [5]. A large trial involving 5,454 patients found that the *H. pylori* eradication rate was 85–94% in patients with good compliance (completing > 80% prescribed medications), but only 39–53% in those with poor compliance [6]. Hence, patient education and interventions promoting treatment adherence are critical for *H. pylori* eradication [7]. Conventional outpatient education for *H. pylori* treatment consists of explaining the reasons for the treatment plan, medication regimen, follow-up schedule, and other relevant factors such as adverse reactions and the importance of treatment completion [7]. Several studies have investigated ways to enhance patient education to improve compliance, such as reminders based on text messages [8] or social media applications [9], telephone follow-up [10–12], and social media communication [13–15].

The “day-to-day (DTD) support model” characterized by daily task-setting and record-keeping behavior has become a popular intervention for chronic diseases, as it promotes goal-setting, habit formation, and active participation in self-management [16, 17]. However, the efficacy of the DTD model for *H. pylori* treatment is not known. Therefore, we developed a DTD model for *H. pylori* management integrated with social media applications. The objective of this study is to evaluate the efficacy of this novel approach in contrast to conventional outpatient education in the treatment of *H. pylori* infection.

## Methods

### Study design

This single-center, prospective, randomized controlled clinical trial was conducted at the First Affiliated Hospital of Nanjing Medical University, Nanjing, Jiangsu Province, China, from September 2021 to December 2022. The inclusion criteria were (a) age, 18–70 years, (b) *H. pylori* infection confirmed by the ^13^ C-urea breath test (UBT), and (c) primary treatment. The exclusion criteria were (a) unfamiliarity with smartphones, (b) allergy to the treatment drugs, (c) treatment with antibiotics, colloidal bismuth pectin, H_2_ receptor inhibitors, or proton pump inhibitors within the previous 4 weeks, (d) serious concurrent diseases, (e) gastrectomy, and (f) pregnancy/lactation. Eligible patients were enrolled after they provided informed consent. The study protocol and informed consent form were approved by the ethics committee of the First Affiliated Hospital of Nanjing Medical University (2021-SR-583). The trial was registered with the Chinese Clinical Trial Registry (ChiCTR2000029021).

### Treatment regimens

Patients were randomized to the DTD or outpatient education (OE) group. Patients in both groups were treated with bismuth-containing quadruple therapy, consisting of esomeprazole 20 mg (Cspc Ouyi Pharmaceutical, Hebei, China), colloidal bismuth pectin capsule 200 mg (Zhendong Anxin Biological Pharmaceutical, Shanxi, China), amoxicillin 1000 mg (Lunan Pharmaceutical, Shandong, China), and clarithromycin 500 mg (Hengrui Pharmaceutical, Jiangsu, China), twice daily for 14 days. In the outpatient clinic, physicians collected the following information: gender, age, body mass index, education, history of smoking/drinking, symptoms before treatment, comorbidity, family history of gastric cancer, lifestyle habits (e.g., washing hands before meals, sharing a toothbrush cup), and knowledge about *H. pylori* infection. To assess patients’ knowledge, the physicians recorded the patients’ answers to the following: Do you know if *H. pylori* is contagious? How many kinds of medication are used to treat *H. pylori*? How long does it take to treat *H. pylori* infection? When should the UBT be rechecked after *H. pylor*i treatment?

### Patient education

All patients received routine outpatient instruction, including verbal and written education. Patients were informed of the importance of *H. pylori* eradication; the dosage, frequency, and adverse effects of the medications; and the review UBT date, and instructed to start taking anti-*H. pylori* drugs from the next day. A uniform instruction manual with notes for the treatment plan and the review UBT date was provided. This manual included the following information: (a) The drugs in the regimen are taken twice a day, and the dose cannot be missed. Esomeprazole and colloidal bismuth pectin must be taken 30 min before breakfast and dinner; the 2 antibiotics must be taken 30 min after breakfast and dinner. (b) The regimen duration is 14 days; treatment interruption should be avoided. (c) Possible adverse reactions include black stools and diarrhea. (d) Alcohol consumption is prohibited during treatment. (e) Follow-up ^13^ C-UBT is required to assess the presence of *H. pylori* infection, at least 1 month after the 14-day treatment regimen has finished. The physician confirmed with the patients if they understood the above information and provided further explanation if required.

### Interventions

**Fig. 1:**
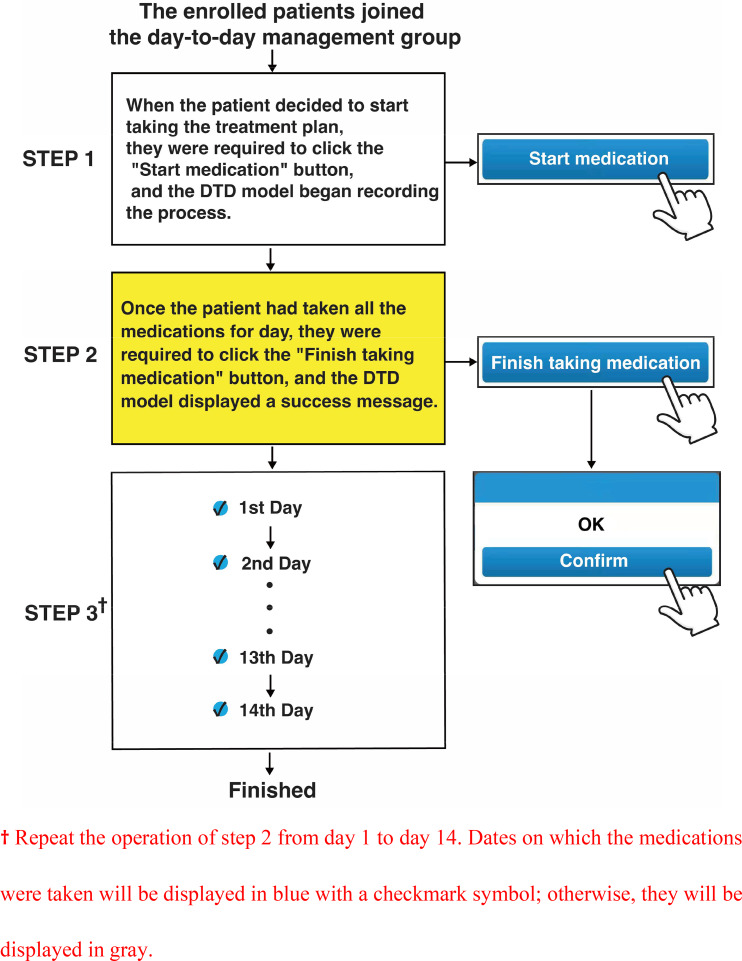
Schematic diagram of the usage instructions for the day-to-day management model

The DTD model was integrated into an official service account of the WeChat platform. After following the account, the enrolled patients logged into the model during their treatment (Supplementary Fig. 1). DTD-group patients underwent DTD management as follows: (a) The patient clicked the “Start medication” button when starting the treatment plan, and the model began recording the treatment process. (b) Once the patient took all the day’s medications, he/she clicked the “Finish taking medication” button, and the model recorded the completion of the day’s treatment and displayed a success message. (c) Dates on which medications were taken were displayed in blue with a checkmark symbol; otherwise, they were displayed in gray. (d) Patients repeated the above process from day 1 to day 14. (e) When the day-14 recording was completed, the model automatically displayed the re-examination time (1 month after the end of the treatment; Fig. 1, Supplementary Fig. 2).

The OE group was not provided with DTD management. On the 14th day of taking medicines, the patients received a telephone call to inquire about the remaining dosages. Two months after treatment completion, the patients were again called to inquire about the review UBT results and to remind patients who had not undergone review UBT. To get as much data as possible to calculate the eradication rate, we again called the patients before the end of the study, and asked about the review UBT results.

### Endpoints

The primary endpoints were medication compliance and follow-up compliance. The secondary endpoint was the *H. pylori* eradication rate. Good medication compliance was defined as taking > 80% of prescribed medications, calculated based on remaining dosages. Follow-up compliance was assessed by recording the review UBT date and calculating the number of days between treatment completion and review UBT (termed “review UBT time”). Completion of the review UBT within 1–2 months after treatment completion was classified as good follow-up compliance. A ^13^ C-UBT value < 4‰ indicated successful *H. pylori* eradication. Treatment-related AEs were also assessed using a standardized questionnaire.

### Statistical analysis

A study reported a follow-up rate of 93.8% in their WeChat intervention group, as compared with 77.6% in their control group (*P* < 0.001) [14]. Assuming an α-error < 0.05, a β-error < 0.1, and a 20% dropout rate, we calculated that at least 122 patients per group would be needed.

Data were analyzed using SPSS *v*26.0. All intention-to‐treat (ITT), modified ITT (MITT), and per-protocol (PP) analyses were performed using the primary and secondary endpoints. In the ITT and MITT analyses, patients with missing data due to exclusion were considered to have failed eradication, poor medication compliance, and poor follow-up compliance. For patients without review UBT, the date of review was defined as February 28, 2023. Continuous variables were presented as median (interquartile range) and analyzed using the Wilcoxon rank-sum test; categorical variables were presented as frequency (percentage) and analyzed using the chi-square or Fisher’s exact test. *P* < 0.05 indicated statistical significance.

## Results

### Baseline characteristics

**Fig. 2:**
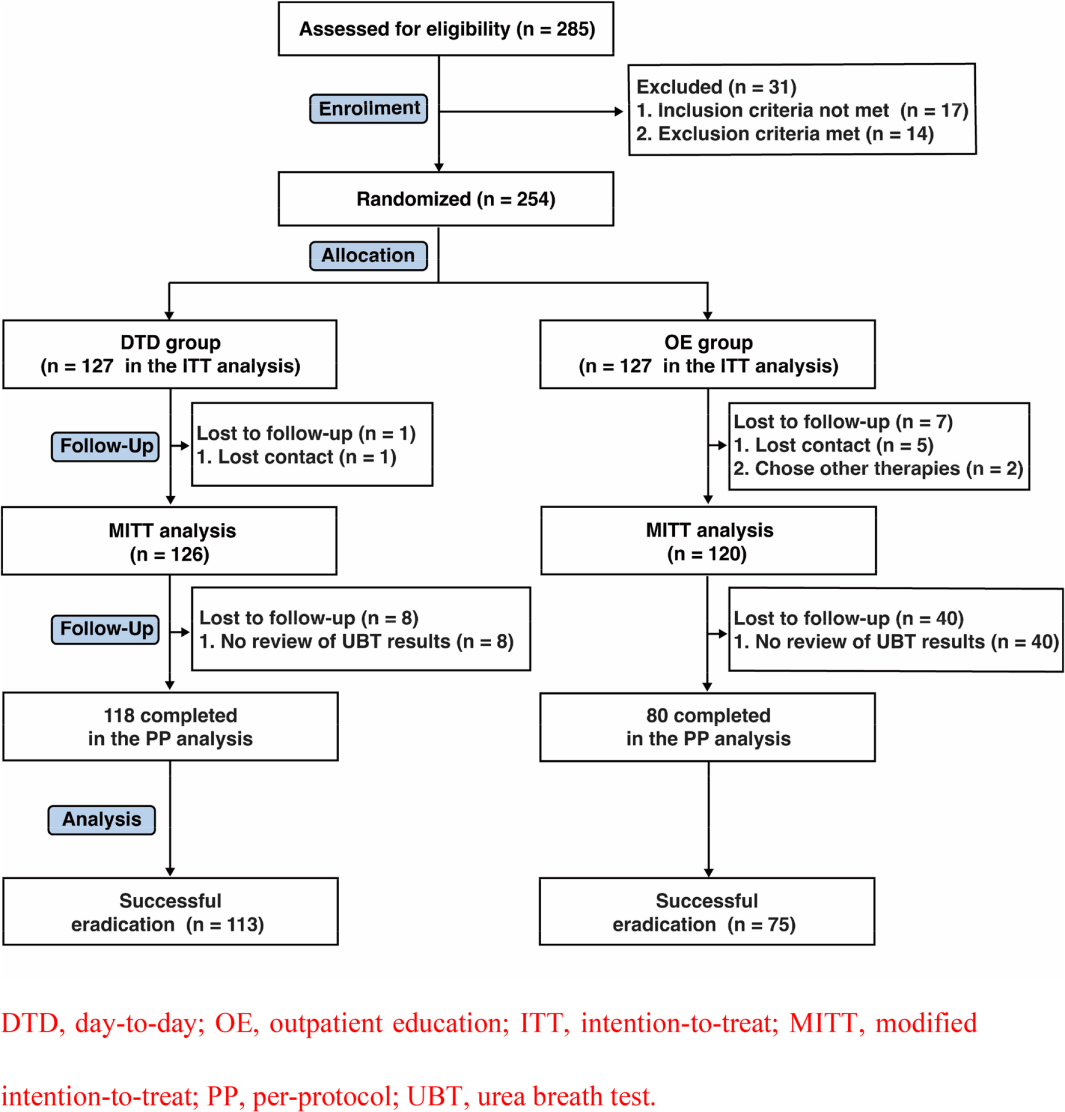
Flow chart of patient selection and study design

In total, 254 patients were randomized to the DTD (127 patients) and OE groups (127 patients; Fig. 2). All patients were included in the ITT analysis. The MITT analysis excluded patients who chose other therapies or were lost to follow-up. Patients lacking review UBT results were excluded from the PP analysis. Finally, 126 and 118 patients from the DTD group were included in the MITT and PP analyses, respectively. In the OE group, 120 and 80 patients were included in the MITT and PP analyses, respectively.

We found no significant between-group differences in the patients’ baseline characteristics, including education, lifestyle habits, and knowledge of *H. pylori* infection (Table 1).

**Table 1:** Baseline characteristics

	DTD group (n = 127)	OE group (n = 127)	*P* value
Gender (male)	62 (48.8)	59 (46.5)	0.706
Age (years)	34.0 (28.0, 44.0)	36.0 (29.0, 51.0)	0.159
Body mass index (kg/m^2^)	22.9 (20.6, 25.4)	22.6 (20.7, 25.2)	0.674
Education status (college or more)	84 (66.1)	81 (63.8)	0.693
Smoking	24 (18.9)	17 (13.4)	0.233
Drinking	38 (29.9)	42 (33.1)	0.589
Symptoms present before treatment	50 (39.4)	52 (40.9)	0.798
Comorbidity	13 (10.2)	18 (14.2)	0.338
Family history of gastric cancer	8 (6.3)	5 (3.9)	0.393
Washing hands before meals	96 (75.6)	102 (80.3)	0.364
Sharing a toothbrush cup	17 (13.4)	25 (19.7)	0.177
Knowing *H. pylori* is contagious	92 (72.4)	92 (72.4)	1.000
Knowing *H. pylori* eradication drugs	24 (18.9)	34 (26.8)	0.135
Knowing *H. pylori* eradication course	29 (22.8)	38 (29.9)	0.200
Knowing the timing of the review UBT	23 (18.1)	23 (18.1)	1.000

DTD, day-to-day; OE, outpatient education; UBT, urea breath test

Values are shown as numbers (percentages) or median (interquartile range)

### Medication compliance

In the ITT analysis, the rates of good medication compliance were 95.3% (121/127; 95% confidence interval [CI]: 91.5–99.0%) in the DTD group and 78.7% (100/127; 95% CI: 71.5–86.0%) in the OE group. The MITT analysis showed that the good medication compliance rates were 96.0% (121/126; 95% CI: 92.6–99.5%) in the DTD group and 83.3% (100/120; 95% CI: 76.6–90.1%) in the OE group. In the PP analysis, the rates of good medication compliance were 95.8% (113/118; 95% CI: 92.1–99.5%) and 87.5% (70/80; 95% CI: 80.1–94.9%) in the DTD and OE group, respectively. Medication compliance significantly differed between the two groups in the ITT, MITT, and PP analyses (*P* < 0.001, *P* = 0.001, and *P* = 0.031, respectively; Table 2).

**Table 2:** Medication compliance

	DTD group	OE group	*P* value
ITT	95.3% (121/127)	78.7% (100/127)	< 0.001
95% CI	91.5–99.0%	71.5–86.0%	
MITT	96.0% (121/126)	83.3% (100/120)	0.001
95% CI	92.6–99.5%	76.6–90.1%	
PP	95.8% (113/118)	87.5% (70/80)	0.031
95% CI	92.1–99.5%	80.1–94.9%	

DTD, day-to-day; OE, outpatient education; ITT, intention-to-treat; MITT, modified intention-to-treat; PP, per-protocol; CI, confidence interval

### Follow-up compliance

The review UBT rate was significantly higher in the DTD group than in the OE group (92.9% vs. 63.0%, *P* < 0.001; Table 3). ITT analysis showed that the good follow-up compliance rates were 81.1% (103/127; 95% CI: 74.2–88.0%) in the DTD group and 44.1% (56/127; 95% CI: 35.3–52.8%) in the OE group. In the MITT analysis, the rates of good follow-up compliance were 81.7% (103/126; 95% CI: 74.9–88.6%) and 46.7% (56/120; 95% CI: 37.6–55.7%) in the DTD and OE groups, respectively. In the PP analysis, the good follow-up compliance rates were 87.3% (103/118; 95% CI: 81.2–93.4%) in the DTD group and 70.0% (56/80; 95% CI: 59.7–80.3%) in the OE group. The follow-up compliance rate significantly differed between the 2 groups in the ITT, MITT, and PP analyses (*P* < 0.001, *P* < 0.001, and *P* = 0.003, respectively; Table 4).

**Table 3:** Rate of review UBT

	DTD group (n = 127)	OE group (n = 127)	*P* value
Review UBT			< 0.001
Yes	118 (92.9)	80 (63.0)	
No	9 (7.1)	47 (37.0)	

DTD, day-to-day; OE, outpatient education; UBT, urea breath test

**Table 4:** Follow-up compliance

	Follow-up compliance rate			Timing of review UBT†		
	DTD group	OE group	*P* value	DTD group	OE group	*P* value
ITT	81.1% (103/127)	44.1% (56/127)	< 0.001	36.0 (31.0, 52.0)	73.0 (35.0, 242.0)	< 0.001
95% CI	74.2–88.0%	35.3–52.8%				
MITT	81.7% (103/126)	46.7% (56/120)	< 0.001	35.5 (31.0, 50.5)	64.5 (35.0, 219.0)	< 0.001
95% CI	74.9–88.6%	37.6–55.7%				
PP	87.3% (103/118)	70.0% (56/80)	0.003	35.0 (31.0, 43.3)	42.0 (32.0, 70.5)	0.001
95% CI	81.2–93.4%	59.7–80.3%				

DTD, day-to-day; OE, outpatient education; UBT, urea breath test; ITT, intention-to-treat; MITT, modified intention-to-treat; PP, per-protocol; CI, confidence interval

Values are shown as numbers (percentages) or median (interquartile range)

†The timing of the review UBT is expressed as the number of days after the completion of the treatment

In the DTD group, the median review UBT time was 36.0 (31.0, 52.0), 35.5 (31.0, 50.5), and 35.0 (31.0, 43.3) days after treatment completion in the ITT, MITT, and PP analyses, respectively. In the OE group, the median review UBT time was 73.0 (35.0, 242.0), 64.5 (35.0, 219.0), and 42.0 (32.0, 70.5) days after treatment completion in the ITT, MITT, and PP analyses, respectively. The median review UBT time significantly differed between the two groups in the ITT, MITT, and PP analyses (*P* < 0.001, *P* < 0.001, and *P* = 0.001, respectively; Table 4).

### H. pylori eradication rate and adverse events

In the ITT analysis, the rates of *H. pylori* eradication were 89.0% (113/127; 95% CI: 83.5–94.5%) in the DTD group and 59.1% (75/127; 95% CI: 50.4–67.7%) in the OE group. The MITT analysis showed that the eradication rates were 89.7% (113/126; 95% CI: 84.3–95.1%) in the DTD group and 62.5% (75/120; 95% CI: 53.7–71.3%) in the OE group. In the PP analysis, the eradication rates were 95.8% (113/118; 95% CI: 92.1–99.5%) and 93.8% (75/80; 95% CI: 88.3–99.2%) in the DTD and OE group, respectively. In the ITT and MITT analyses, the eradication rates were significantly higher in the DTD group than in the OE group (both *P* < 0.001). However, no significant difference in the eradication rate was found in the PP analysis (*P* = 0.529; Table 5). The incidence of AEs was comparable between the DTD and OE groups (25.4% vs. 28.3%, *P* = 0.603; Table 6).

**Table 5:** Rate of *H. pylori* eradication

	DTD group	OE group	*P* value
ITT	89.0% (113/127)	59.1% (75/127)	< 0.001
95% CI	83.5–94.5%	50.4–67.7%	
≤ 40 years	92.2% (83/90)	57.7% (45/78)	< 0.001
> 40 years	81.1% (30/37)	61.2% (30/49)	0.047
MITT	89.7% (113/126)	62.5% (75/120)	< 0.001
95% CI	84.3–95.1%	53.7–71.3%	
≤ 40 years	92.2% (83/90)	61.6% (45/73)	< 0.001
> 40 years	83.3% (30/36)	63.8% (30/47)	0.049
PP	95.8% (113/118)	93.8% (75/80)	0.529†
95% CI	92.1–99.5%	88.3–99.2%	
≤ 40 years	96.5% (83/86)	95.7% (45/47)	1.000†
> 40 years	93.8% (30/32)	90.9% (30/33)	1.000†

DTD, day-to-day; OE, outpatient education; ITT, intention-to-treat; MITT, modified intention-to-treat; PP, per-protocol; CI, confidence interval; †, Fisher’s exact test

**Table 6:** Adverse events in the DTD group and the OE group

	DTD group (n = 126)	OE group (n = 120)	*P* value
Total	32 (25.4)	34 (28.3)	0.603
Nausea/vomiting	2 (1.6)	4 (3.3)	0.437†
Abnormal taste	22 (17.5)	18 (15.0)	0.601
Diarrhea	7 (5.6)	5 (4.2)	0.613
Abdominal pain	3 (2.4)	2 (1.7)	1.000†
Skin rash	1 (0.8)	2 (1.7)	0.614†
Others	2 (1.6)	5 (4.2)	0.272†
Discontinued drugs because of adverse events	2 (1.6)	3 (2.5)	0.678†

Adverse events were evaluated in modified intention-to-treat

DTD, day-to-day; OE, outpatient education; †, Fisher’s exact test

## Discussion

Numerous factors contribute to compliance with *H. pylori* treatment, including treatment complexity and duration, physician motivation, patient education, and effective medication regimens [18]. Two meta-analyses showed that enhanced patient education significantly improved *H. pylori* eradication rates and compliance [19, 20]. Compared to telephone follow-up, social media communications offer several advantages for clinical follow-up, such as convenience, real-time communication allowing for timely feedback and problem resolution, the ability to save and back up information, and multimodal communication, including text, images, and videos, to better meet the diverse communication needs of patients and doctors.

WeChat is a popular free instant messaging and social media platform in China. It can be used for one-on-one conversations (WeChat messages) as well as group messages (WeChat groups). Several studies have demonstrated that intervention groups utilizing WeChat features, such as messages or groups, improved eradication rates and medication adherence, as compared to control groups [13, 14]. However, Lin et al. reported no significant difference in *H. pylori* eradication rates or compliance between WeChat-based intervention and conventional patient education [15]. To investigate the causes of this inconsistency, we examined the differences in the *H. pylori* eradication rates between the conventional OE groups in the above studies. Luo et al. reported a success rate of 63.1% and good adherence in 54% of control patients (who received quadruple therapy), but did not report the type of analysis used (ITT or PP) or the details of the treatment plan [13]. In the study by Ma et al., PP analysis showed that bismuth-containing quadruple therapy including amoxicillin and furazolidone achieved an eradication rate of 78.6% in the control group, with a follow-up rate of 77.6% [14]. A success rate of < 80% is considered low for *H. pylori* treatment regimens [21, 22]. Notably, a meta-analysis of 18 studies found that furazolidone-containing bismuth-containing quadruple therapy had an eradication rate of 92.9% (95% CI: 90.7–95.1%) in the PP analysis [23], and resistance of *H. pylori* to furazolidone is rare in China [24]. Therefore, when initiating treatment for *H. pylori* infection, physicians should prioritize selecting a superior effective regimen with a high cure rate in the local population [25, 26]. Lin et al. used bismuth-containing quadruple therapy with amoxicillin and clarithromycin, and achieved an eradication rate of 88.2% in the control group [15]. When the eradication rate is already high in the OE group, no significant difference may be detected between the OE and intervention groups [15, 27]. Similar results were observed in our study in the PP analysis; the *H. pylori* eradication rate in the control group (93.8%) did not significantly differ from that in our intervention group (95.8%), and no differences were found in the subgroup analysis based on age. Sun et al. reported that daily medication reminders based on a WeChat mini-program improved patient compliance but not the *H. pylori* eradication rate (82.9% in the control group in PP analysis) [9]. Our study and the above study used the same treatment regimen; hence, the higher success rate in our control group may be attributed to differences in outpatient education, which consisted of only verbal education in the above study, and verbal education combined with detailed written instructions in our study. This demonstrates the significance of incorporating diverse and sufficient educational methods in physician-patient interactions.

WeChat messaging and group chats (as well as telephone calls and text messages) rely on physician-patient communication, and require doctors to be fully engaged throughout the entire process, often utilizing their work and personal time [8, 10, 11]. Thus, it is important to explore alternative effective intervention methods that can alleviate doctors’ workload, particularly during follow-up. The WeChat-based mini-app developed by Sun et al. has a reminder function, which requires patients to confirm medication intake, and another reminder is sent after a certain period if the initial reminder is ignored [9]. However, the entire process is a passive behavior for patients. In recent years, the DTD management method has become a popular tool for individuals to track and maintain good habits through daily motivation, emphasizing the role of personal initiative in habit formation. Therefore, we developed a self-management DTD model for *H. pylori* treatment. The model allows patients to mark a completion “flag” after taking all prescribed medications for the day from day 1 to day 14, serving as a motivation that they have completed their daily treatment plan. The habit-formation process could be a valuable tool in promoting positive health behaviors [28] and improving therapy adherence [29]. Subgroup analyses of our study revealed no significant differences in the compliance rates based on age and education level in the DTD model (Supplementary Table 1). This suggests that the model is accessible and easily implementable for a broad population who have the WeChat platform.

All patients undergoing *H. pylori* therapy are recommended to be reviewed after 4–6 weeks to confirm *H. pylori* eradication [1]. Failure to perform timely follow-up can affect treatment effectiveness, lead to antibiotic resistance [26], and increase the exposure risk of patients’ close contacts [3]. Upon completion of the 14-day regimen, the DTD model automatically displayed the date of the follow-up UBT (defaulting to 1 month after treatment completion). Our study showed significant differences in the rate of review UBT, follow-up compliance, and median review UBT time between the DTD and OE groups. These results collectively suggest that the DTD model effectively improved follow-up compliance without requiring physician involvement.

To the best of our understanding, we are the first to combine the use of anti-*H. pylori* drugs with the DTD model for *H. pylori* treatment, allowing patients to achieve medication goals by forming habits and self-monitoring the entire medication process, without the need for physician intervention throughout the medication process. The novel strategy may inspire clinician to develop similar DTD model as a behavioral intervention in management of *H. pylori* treatment. However, the study has several limitations. First, this was a single-center study; larger sample sizes are needed to validate our findings. Second, *H. pylori* drug-sensitivity testing was not performed on patients receiving primary treatment in either group, and while the groups were randomized, there is no assurance that they were not statistically different in terms of amoxicillin and clarithromycin resistance rates. Third, the requirement for smartphone usage may limit the generalizability of this model, particularly in older individuals or those with poor economic conditions; re-education interventions may be more appropriate for such people, though further research is needed to confirm this hypothesis.

In conclusion, adequate outpatient education is crucial for ensuring compliance, which involves taking medications as directed, attending follow-up appointments, and making necessary lifestyle changes. The DTD model improved patient compliance without needing continual physician involvement. Our findings suggest that the DTD model may be a useful tool for physicians managing *H. pylori* treatment, particularly in cases with limited outpatient education.

## Electronic supplementary material

Below is the link to the electronic supplementary material.

Supplementary Material 1

Supplementary Material 2

Supplementary Material 3

## Data Availability

All data are available without restriction. Researchers can obtain data by contacting the corresponding author. All data generated or analyzed during this study are included in this published article.

## Abbreviations

H. pylori: Helicobacter pylori
DTD: Day-to-day
OE: Outpatient education
UBT: Urea breath test
ITT: Intention-to-treat
MITT: Modified intention-to-treat
PP: Per protocol
