# Supplementary figures and images for: A day-to-day management model improves patient compliance to treatment for Helicobacter pylori infection: a prospective, randomized controlled study
Source: Gut Pathog. 2023 Jul 31;15:38. doi: 10.1186/s13099-023-00556-x (PMC10388557; doi:10.1186/s13099-023-00556-x)

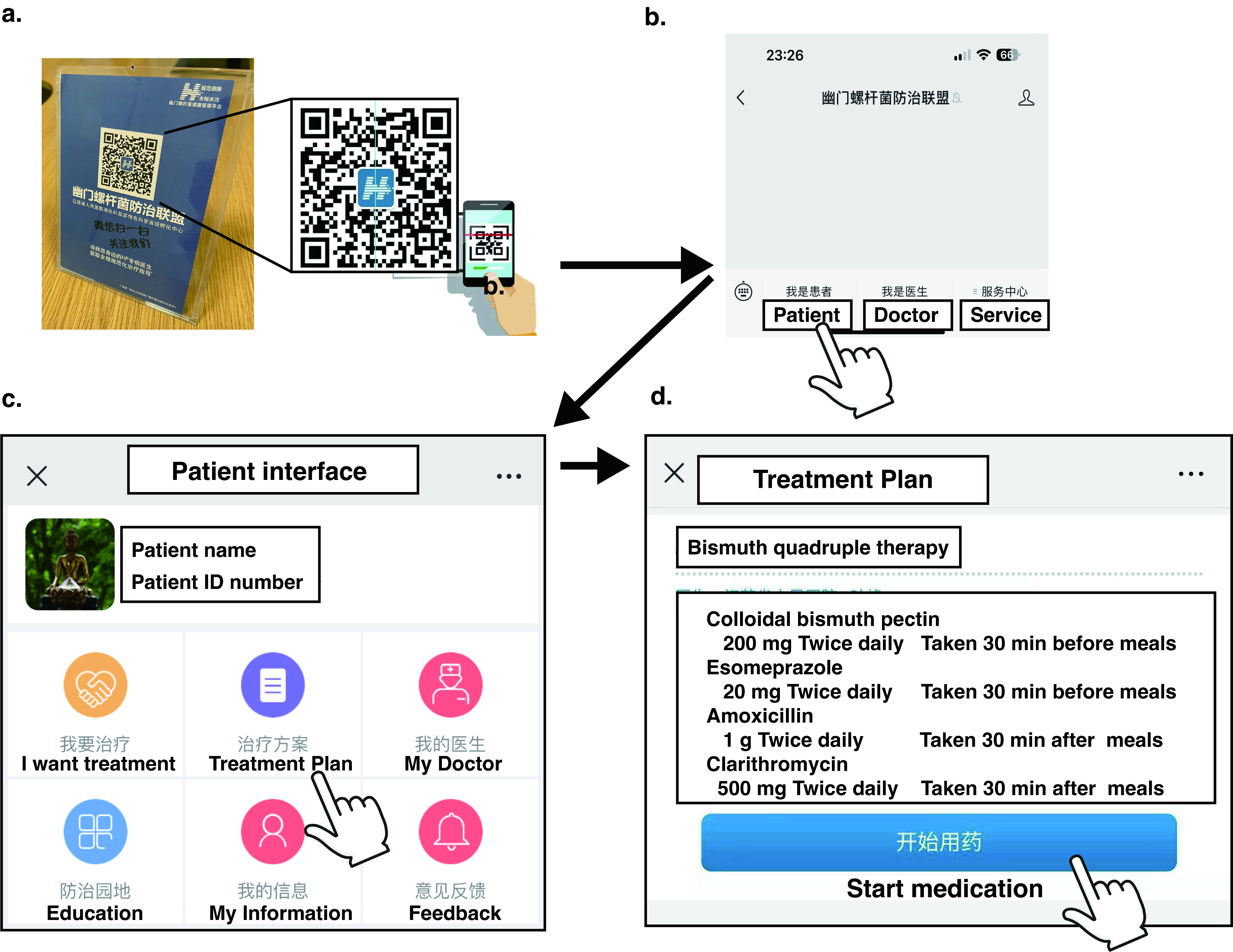

Supplement: Supplementary file 1 — Supplementary Material 1 [file 13099_2023_556_MOESM1_ESM.tif]

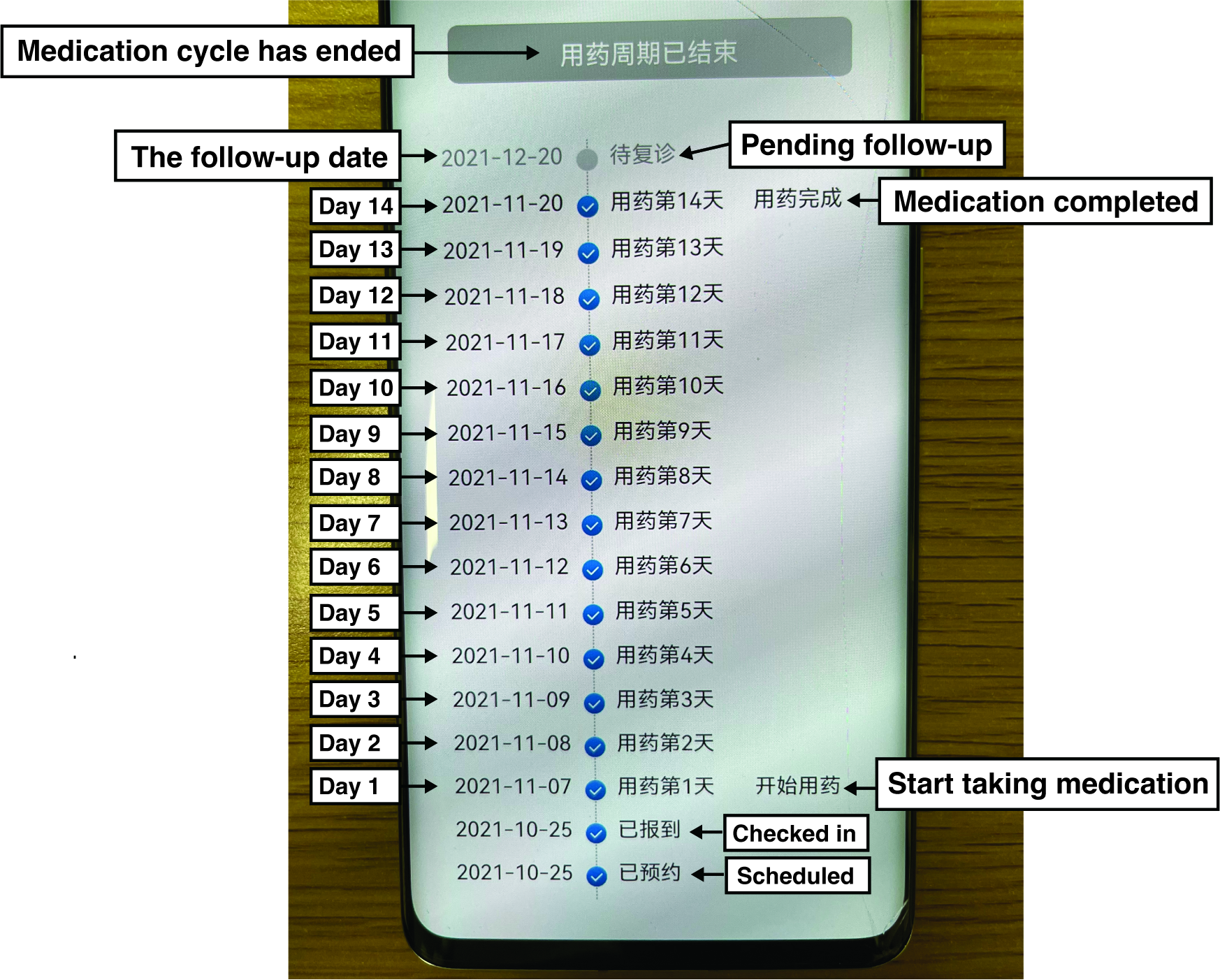

Supplement: Supplementary file 2 — Supplementary Material 2 [file 13099_2023_556_MOESM2_ESM.tif]
