# Supplementary material for: A day-to-day management model improves patient compliance to treatment for Helicobacter pylori infection: a prospective, randomized controlled study
Source: Gut Pathog. 2023 Jul 31;15:38. doi: 10.1186/s13099-023-00556-x (PMC10388557; doi:10.1186/s13099-023-00556-x)
Supplement: Supplementary file 3 — Supplementary Material 3 [file 13099_2023_556_MOESM3_ESM.docx]

**Supplementary Figure 1. Use of the DTD model through the service account of WeChat.**

**
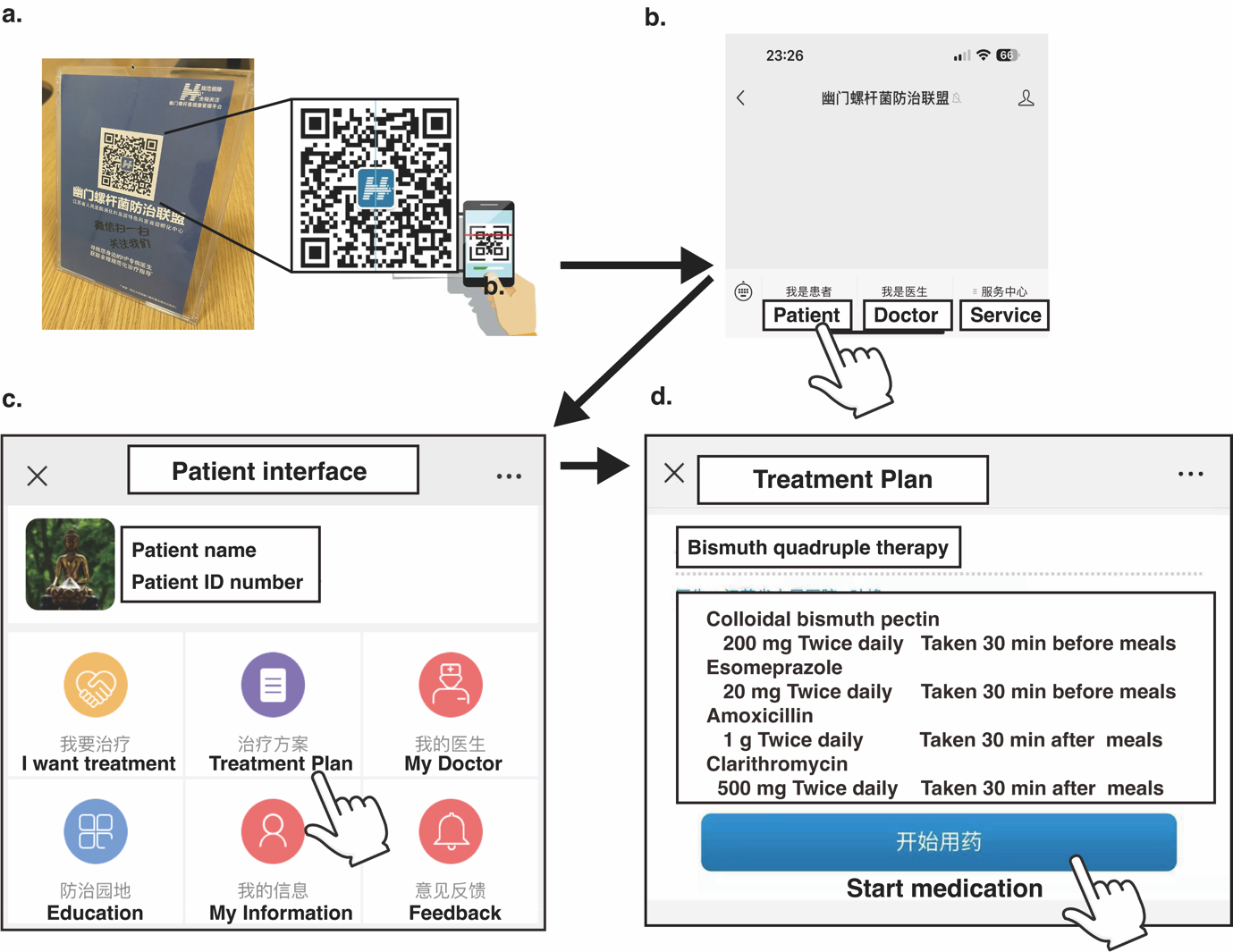
**

(a) Patients can access the WeChat official account by scanning the QR code. (b, c) After selecting “Patient” and completing the registration, patients can access the patient interface. In the “I want treatment” section, patients can make an online appointment with a physician, finalizing the “Scheduled” step. On the visit day, patients scan the physician’s QR code to complete the “check-in” step. (d) After the visit, the treatment plan is available in the “Treatment plan” section of the interface. Upon initiating their medication regimen, patients can click “Start Medication” to begin the tracking process.

**Supplementary Figure 2. Photograph showing the treatment course of an *H. pylori*-positive patient in the DTD group.**

**
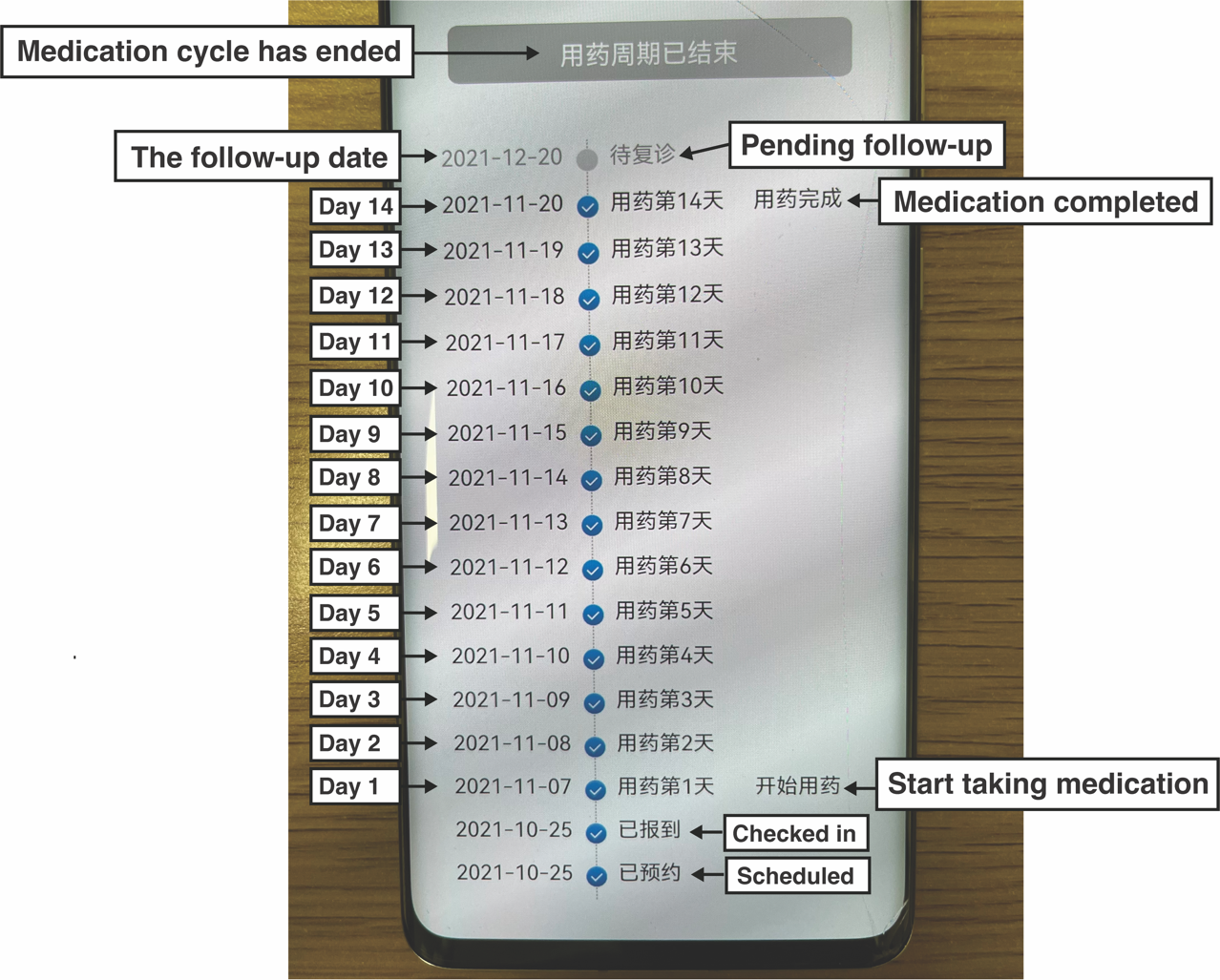
**

The image shows that the patient was enrolled on November 6th, 2021 and began taking medication on November 7th, 2021. The patient used the day-to-day (DTD) management model to check in each day from the 1^st^ day to the 14^th^ day. On the last day, the model displayed the “Medication completed” notification and the follow-up date of December 20^th^, 2021. The check-in button will display “Medication cycle has ended.” The photo was taken during the patient’s follow-up visit.

**Supplementary Table 1. Subgroup analysis of medical compliance in the DTD group based on patients’ age and education**

|  | **Good compliance**  **(n = 121)** | **Poor compliance**  **(n = 6)** | ***P* value** |
| --- | --- | --- | --- |
| Age (years) |  |  | 0.671† |
| ≤40 | 85 (70.2) | 5 (83.3) |  |
| >40 | 36 (29.8) | 1 (16.7) |  |
| Education level |  |  | 0.663† |
| College and above | 79 (65.3) | 5 (83.3) |  |
| Below college | 42 (34.7) | 1 (16.7) |  |

DTD, day-to-day; †, Fisher’s exact test.
